# *Trypanosoma brucei* aquaglyceroporin 2 is a high-affinity transporter for pentamidine and melaminophenyl arsenic drugs and the main genetic determinant of resistance to these drugs

**DOI:** 10.1093/jac/dkt442

**Published:** 2013-11-13

**Authors:** Jane C. Munday, Anthonius A. Eze, Nicola Baker, Lucy Glover, Caroline Clucas, David Aguinaga Andrés, Manal J. Natto, Ibrahim A. Teka, Jennifer McDonald, Rebecca S. Lee, Fabrice E. Graf, Philipp Ludin, Richard J. S. Burchmore, C. Michael R. Turner, Andy Tait, Annette MacLeod, Pascal Mäser, Michael P. Barrett, David Horn, Harry P. De Koning

**Affiliations:** 1 Institute of Infection, Immunity and Inflammation, College of Medical, Veterinary and Life Sciences, University of Glasgow, Glasgow, UK; 2 Wellcome Trust Centre for Molecular Parasitology, University of Glasgow, Glasgow, UK; 3 Department of Medical Biochemistry, University of Nigeria, Enugu Campus, Enugu, Nigeria; 4 London School of Hygiene and Tropical Medicine, London, UK; 5 Swiss Tropical and Public Health Institute, Basel, Switzerland; 6 University of Basel, Basel, Switzerland

**Keywords:** drug transport, protozoan, parasite, resistance mutation, aquaporin

## Abstract

**Objectives:**

*Trypanosoma brucei* drug transporters include the *TbAT1*/P2 aminopurine transporter and the high-affinity pentamidine transporter (HAPT1), but the genetic identity of HAPT1 is unknown. We recently reported that loss of *T. brucei* aquaglyceroporin 2 (*TbAQP2*) caused melarsoprol/pentamidine cross-resistance (MPXR) in these parasites and the current study aims to delineate the mechanism by which this occurs.

**Methods:**

The *TbAQP2* loci of isogenic pairs of drug-susceptible and MPXR strains of *T. brucei* subspecies were sequenced. Drug susceptibility profiles of trypanosome strains were correlated with expression of mutated *TbAQP2* alleles. Pentamidine transport was studied in *T. brucei* subspecies expressing *TbAQP2* variants.

**Results:**

All MPXR strains examined contained *TbAQP2* deletions or rearrangements, regardless of whether the strains were originally adapted *in vitro* or *in vivo* to arsenicals or to pentamidine. The MPXR strains and AQP2 knockout strains had lost HAPT1 activity. Reintroduction of *TbAQP2* in MPXR trypanosomes restored susceptibility to the drugs and reinstated HAPT1 activity, but did not change the activity of *TbAT1*/P2. Expression of *TbAQP2* sensitized *Leishmania mexicana* promastigotes 40-fold to pentamidine and >1000-fold to melaminophenyl arsenicals and induced a high-affinity pentamidine transport activity indistinguishable from HAPT1 by *K*_m_ and inhibitor profile. Grafting the *TbAQP2* selectivity filter amino acid residues onto a chimeric allele of *AQP2* and *AQP3* partly restored susceptibility to pentamidine and an arsenical.

**Conclusions:**

TbAQP2 mediates high-affinity uptake of pentamidine and melaminophenyl arsenicals in trypanosomes and *TbAQP2* encodes the previously reported HAPT1 activity. This finding establishes TbAQP2 as an important drug transporter.

## Introduction

The protozoan parasite *Trypanosoma brucei* is the aetiological agent of human African trypanosomiasis (HAT or sleeping sickness). The subspecies *T. b. gambiense* and *T. b. rhodesiense* are responsible for West African and East African sleeping sickness, respectively, and *T. b. brucei* is one of the pathogens that cause animal African trypanosomiasis, a wasting disease of livestock. Despite the recent introduction of nifurtimox/eflornithine combination therapy for the late, cerebral stage of HAT,^1^ there is an urgent need for new drugs, driven in part by resistance to the diamidines, phenanthridines and melaminophenyl arsenicals (MPAs) that have been the central pillars of African trypanosomiasis treatment for decades.^2^ An understanding of the mechanisms of resistance, and particularly of cross-resistance, is of great importance. Firstly, molecular markers are required to study the epidemiology of resistance, particularly as phenotypic assessment in primary clinical/veterinary isolates is impossible for many species of African trypanosome and there is an unresolved debate about the extent of treatment failure versus genuine resistance, especially with respect to melarsoprol.^3^ Secondly, in the absence of new drugs we need to make best use of the treatments available and, for this, insight into resistance mechanisms and levels of cross-resistance is essential. Importantly, new drug development must take into account the resistance mechanisms to the current drugs, in order to avoid cross-resistance.

Melarsoprol/pentamidine cross-resistance (MPXR) is a well-known phenomenon in HAT and was first noted by Rollo and Williamson in 1951;^4^ although its causes have never been completely resolved, it has long been clear this is linked to reduced drug accumulation.^5–7^ The first drug transporter identified in trypanosomes was the P2 adenosine/adenine transporter, which was initially implicated in melarsoprol uptake^8^ and subsequently also in diamidine transport;^9–11^ the gene was designated *TbAT1*.^12^ All protozoan nucleoside and nucleobase transporters identified to date have been of the equilibrative nucleoside transporter family.^13^ Although the evidence of diamidine and arsenical transport by *TbAT1*/P2 has become incontrovertible, it has become equally clear that this transporter mediates only part of the uptake and that this proportion is different for different diamidines in particular, as deletion of the *TbAT1* gene led to a high level of resistance to the veterinary diamidine diminazene aceturate^14^ and the newer clinical candidates furamidine and CPD0801,^15^ but only to a relatively minor loss of susceptibility to MPAs and pentamidine.^14,16^ Two additional, adenosine-insensitive pentamidine transport activities were detected and functionally characterized in *T. b. brucei*: a high-affinity pentamidine transporter (HAPT1) and a low-affinity pentamidine transporter (LAPT1).^17,18^ HAPT1 was additionally found to be the secondary transporter for the arsenical drugs, with the loss of both the P2 and HAPT1 activities simultaneously leading to high-level MPXR.^16,19^ Despite the HAPT1 and LAPT1 activities having been first characterized over a decade ago,^17^ the genes encoding these transporters remained unknown.

Recently, we reported that the aquaglyceroporin TbAQP2 controls MPXR in *T. b. brucei*.^20^ Aquaporins (AQPs) are major intrinsic proteins (MIPs) that are present in virtually every organism and are commonly implicated in osmotic balance and, in the case of aquaglyceroporins, in the bidirectional flux of some small, usually uncharged solutes, such as glycerol and urea.^21^ AQPs have attracted increasing pharmacological interest because of their important roles in many human physiological and pathophysiological processes, including cancer, post-traumatic brain oedema, glaucoma and epilepsy.^22,23^ Further pharmacological interest in AQPs emerged when it became clear that these water channels can also mediate the uptake of a wider array of molecules, including some that are cytotoxic and display antimicrobial activity.^24^ Some AQPs, including *Leishmania major* AQP1, transport antimony and arsenic, most likely in the form of As(OH)_3_ and Sb(OH)_3_, which structurally resemble glycerol.^25,26^ This has attracted much attention, because pentavalent antimonials such as Glucantime and Pentostam, which are activated to a form of Sb(III), are a first-line treatment for leishmaniasis.

*T. brucei* members of the AQP family are classified functionally^27,28^ and phylogenetically^29^ as aquaglyceroporins. They are closely related to LmAQP1 and human aquaglyceroporins, including hAQP9, which reportedly allows the uptake of a wide variety of uncharged solutes, including carbamides, polyols, purines and pyrimidines.^30^ The three *T. b. brucei* AQPs appear to have very similar permeation patterns, mediating the uptake of glycerol, dihydroxyacetone, ribitol and urea.^27^ However, only TbAQP2 was implicated in MPXR, with the re-expression of TbAQP3 in an *aqp2/aqp3* null line having no effect on drug susceptibility.^20^

Here, we report that loss of the wild-type *TbAQP2* open reading frame (ORF) was observed in all MPXR strains (*T. b. brucei*, *T. b. gambiense* and *T. b. rhodesiense*), whether they were selected for resistance to MPAs or pentamidine, including strains selected *in vivo* and able to be transmitted by tsetse flies. Based on our detailed genetic, pharmacological and kinetic analysis, we conclude that *TbAQP2* encodes the HAPT1 activity and that loss of AQP2 function is sufficient and likely required for high-level MPXR.

## Materials and methods

### Trypanosome strains and culture

Bloodstream-form *T. b. brucei*, strain Lister 427 (s427; MiTat 1.2/BS221), and its derivatives were maintained as previously described.^16^ Several derivative lines were used: *tbat1*^−*/*−^,^14^ B48,^16^ 2T1,^31^ *aqp2/aqp3* null strains^32^ and P1000 cells (this paper). Procyclic-form *T. b. gambiense* STIB 386 wild-type and Cymelarsan-resistant (386MR) lines, and *T. b. brucei* STIB 247 wild-type and Cymelarsan-resistant (247MR) lines were grown as described previously.^33^ The P1000 line was generated by further subculturing of bloodstream forms of the B48 line in incrementally increasing concentrations of pentamidine, starting at 75 nM, until the trypanosomes proliferated in 1 μM pentamidine. This process took almost a year of continuous *in vitro* adaptation (Figure S1a, available as Supplementary data at *JAC* Online), which was presumably genetic in nature as the resistance phenotype has proven to be completely stable even after storage in liquid nitrogen or transformation to procyclic cells. There was no apparent *in vitro* growth defect associated with the P1000 adaptations (Figure S1b, available as Supplementary data at *JAC* Online). The STIB 900 line is *T. b. rhodesiense*, originally isolated from a human patient in Tanzania, and was adapted *in vitro* for resistance to pentamidine (STIB 900-P) or melarsoprol (STIB 900-M).^34^

### Leishmania strains and culture

*Leishmania mexicana* promastigotes of strain MNYC/BZ/62/M379^35^ were cultured in HOMEM medium (Invitrogen) supplemented with 10% fetal bovine serum at 25°C exactly as described for *L. major* promastigotes.^36^ Promastigotes were passed to fresh culture medium or used for analysis when in mid-log culture.

### Expression of aquaglyceroporins in T. b. brucei cell lines

*AQP2* was expressed in the B48 and P1000 lines by modification of the expression vector pHD1336^37^ to give pHDK21. This plasmid was digested with NotI prior to transfection into trypanosomes. The pRPa^iGFPx^ construct^38^ was modified to express either the *AQP2* or *AQP2-3* chimera genes and was digested with AscI prior to transfection. Primer sequences are given in Table S1 (available as Supplementary data at *JAC* Online). The *AQP3* and *AQP2-3* genes with their selectivity region altered to that of AQP2^20^ were synthesized by GenScript (New Jersey, USA) for insertion into pRPa^iGFPx^,^38^ to give N-terminally tagged proteins. The constructs were digested with AscI prior to transfection. B48, P1000 or *aqp2/aqp3* null strains were washed in Human T Cell Nucleofector Solution for transfection using the appropriate cassette with an Amaxa Nucleofector as described previously.^39^ Transfectants were grown and cloned out by limiting dilution in standard HMI-9/FBS containing the relevant antibiotic (5 μg/mL blasticidin for pHDK21 and 2 μg/mL hygromycin for pRPa^AQP2^/pRPa^AQP2-3^).

### Genome sequencing of STIB 900 lines

Whole genome sequencing of the three *T. b. rhodesiense* lines STIB 900, STIB 900-M and STIB 900-P was carried out by 454 Life Sciences (Branford, CT, USA) on the Genome Sequencer FLX Titanium, performing two shotgun runs per line. All the high-quality reads were mapped to the reference genome *T. b. brucei* 927^40^ from EBI-EMBL (version October 2011) using SMALT (www.sanger.ac.uk/resources/software/smalt). Consensus sequence and variants were identified with SAMtools^41^ and inspected using Artemis.^42^

### Sequencing of AQP2 and AQP3 genes in drug-resistant lines

The *AQP2* and *AQP3* genes were sequenced from the drug-resistant lines as well as from their respective wild-type lines. The genes were amplified from genomic DNA using a proofreading polymerase and ligated into the pGEM-T Easy subcloning vector and sequenced using standard procedures. The primers used for amplification of the various genes are given in Table S1 (available as Supplementary data at *JAC* Online).

### Cellular localization of chimeric AQP2/3

Localization of green fluorescent protein (GFP)-coupled chimeric AQP2/3 (GFP-chAQP2/3) and western blot with anti-GFP antiserum were performed exactly as described for wild-type TbAQP2 and TbAQP3.^20^ Nuclear staining was performed with 4′,6-diamidino-2-phenylindole (DAPI).

### Transport assays

Transport assays with procyclic-^43^ and bloodstream-form trypanosomes,^44,45^ and *L. mexicana* promastigotes,^36,46^ were performed as described previously. Cultures were harvested at the mid-log growth phase and washed into assay buffer (AB; 33 mM HEPES, 98 mM NaCl, 4.6 mM KCl, 0.55 mM CaCl_2_, 0.07 mM MgSO_4_, 5.8 mM NaH_2_PO_4_, 0.3 mM MgCl_2_, 23 mM NaHCO_3_ and 14 mM glucose, pH 7.3) at a final concentration of 10^8^ cells/mL. Transport was initiated by the addition of 100 μL cells to 100 μL of a solution of the appropriate radiolabel in AB layered over oil [7 : 1 dibutylphthalate/mineral oil (v/v); Sigma–Aldrich, St Louis, MO, USA] and terminated by the addition of an ice-cold solution of 1 mL of unlabelled permeant and immediate centrifugation through the oil layer. Radioactivity in the cell pellet was determined by liquid scintillation counting and corrected for non-specific association of radiolabel with the cells as described previously.^45^ All experiments were performed in triplicate on at least three independent occasions and analysed using the appropriate linear and non-linear regression equations in GraphPad Prism 5.0. [^3^H]pentamidine (3.26 TBq/mmol; product TRQ40084, batch 1) was custom synthesized by Amersham using tritium gas, producing a general labelling of the pentamidine molecule.

### Drug susceptibility assays

Drug susceptibilities of the bloodstream-form trypanosomes^47^ and *L. mexicana* promastigotes^48^ were determined using the Alamar blue assay exactly as described previously, measuring fluorescence in 96-well plates with a FLUOstar Optima (BMG Labtech, Durham, NC, USA) at wavelengths of 544 nm for excitation and 620 nm for emission. EC_50_ values were calculated by non-linear regression using an equation for a sigmoidal dose–response curve with variable slope (GraphPad Prism 5.0; GraphPad Software Inc., San Diego, CA, USA).

### Heterologous expression of T. brucei AQPs in Leishmania promastigotes

*TbAQP2* and *TbAQP3* were amplified from genomic DNA by PCR using Phusion polymerase (Thermo Scientific) and subcloned into the pNUS vector for expression in *Leishmania*.^49^ The construct was verified by sequencing before transfection into *L. mexicana* promastigotes using an Amaxa Nucleofector (program U-33).

## Results

### Status of drug transporters and AQP2 in isogenic susceptible/resistant trypanosome pairs

The study by Baker *et al*.^20^ established a clear link between TbAQP2 and MPXR in the s427/B48 isogenic pair of *T. b. brucei*. The B48 line was produced from the *TbAT1*-KO line (derived from s427 by targeted deletion of *TbAT1*)^14^ followed by *in vitro* exposure to pentamidine.^16^ For *TbAQP2* to be confirmed as a general genetic marker for MPXR in trypanosomes, however, it is essential that this link be upheld in further isogenic pairs showing MPXR, particularly where resistance has been induced (i) *in vivo* or (ii) to the arsenical component rather than pentamidine and (iii) in human-infective trypanosome subspecies. We thus widened our investigations to the strains described in Table 1, which lists a number of well-described isogenic strains with the desired characteristics. These include strains of both human-infective subspecies, *T. b. gambiense* and *T. b. rhodesiense*, in addition to the closely related animal parasite *T. b. brucei*. The strains were adapted by drug exposure *in vivo* or *in vitro*, by exposure to an MPA compound (melarsoprol or Cymelarsan, its water-soluble derivative) or pentamidine. Some were shown to be transmissible by tsetse flies and to mate in this vector.^33^ In all of these highly resistant strains, the *TbAT1*/P2 transport activity is known to be deleted, non-expressed or mutated (Table 1 and references therein) and it is believed that this explains part, but crucially not all, of the resistant phenotype.

**Table 1.** Overview of trypanosome strains used in this study

Strain	Subspecies	Susceptibility^a^		Adapted from	Resistance induction	Infectivity		Transport activity			References
		pentamidine	arsenical			rodents	tsetse	P2	HAPT1	LAPT1	
Lister 427	*T. b. brucei*	+++	+++	NA	NA	√	√^b^	√	√	√	^14,16,17^
2T1	*T. b. brucei*	+++	+++	Lister 427	NA	unkn	unkn	√	√	√	^31,20^
*aqp2*/*aqp3* null	*T. b. brucei*	+/−	+	2T1	NA	unkn	unkn	√	NP	√	^20^
TbAT1-KO	*T. b. brucei*	++	++	Lister 427	TGD	√	unkn	NP	√	√	^14^
B48	*T. b. brucei*	−	+/−	TbAT1-KO	*in vitro*, pentamidine	√	unkn	NP	NP	√	^16^
P1000	*T. b. brucei*	−−−	+/−	B48	*in vitro*, pentamidine	unkn	unkn	NP	NP	√	—
STIB 247	*T. b. brucei*	+++	+++	NA	NA	√	√	√	√	√	^33,57,52^
STIB 247MR	*T. b. brucei*	+/−	−	STIB 247	*in vivo*, Cymelarsan	√	√	NP	NP	√	^33,57,52^
STIB 386	*T. b. gambiense*	+++	++	NA	NA	√	√	√	√	√	^33,57,52^
STIB 386MR	*T. b. gambiense*	+/−	−	STIB 386	*in vivo*, Cymelarsan	√	√	NP	NP	√	^33,57,52^
STIB 900	*T. b. rhodesiense*	+++	+++	NA	NA	√	unkn	√	unkn	unkn	^56^
STIB 900-M	*T. b. rhodesiense*	−	−	STIB 900	*in vitro*, Cymelarsan	√	unkn	NP	unkn	unkn	^56^
STIB 900-P	*T. b. rhodesiense*	−	+/−	STIB 900	*in vitro*, pentamidine	√	unkn	mut^c^	unkn	unkn	^56^

√, present; NP, not present; NA, not applicable; unkn, unknown; TGD, targeted gene deletion of the TbAT1 gene creating a *tbat1* null line; MR or -M, resistance induced to melaminophenyl arsenicals (melarsoprol and/or Cymelarsan); -P, resistance induced to pentamidine.

^a^Susceptibility to pentamidine or arsenical drugs is indicated on a relative scale as highly sensitive (+++) ranging to highly resistant to pentamidine or melaminophenyl arsenicals (−−−).

^b^The clone used in this paper is not tsetse transmissible but other clones of s427 have been shown to infect tsetse flies.^34^

^c^Bernhard *et al*.^56^ reported that STIB 900-P contained a wild-type *TbAT1* gene; later analysis revealed that in fact the *TbAT1* open reading frame contains one coding mutation (G1288C, leading to Gly^430^ → Arg).

**Table 2.** Kinetic parameters of high-affinity and low-affinity pentamidine transport in 247 and 386 procyclics

	Pentamidine		Propamidine	Pentamidine	
	HAPT1 *K*_m_ (μM)	HAPT1 *V*_max_ (pmol/10^7^ cells/s)	HAPT1 *K*_i_ (μM)	LAPT1 *K*_m_ (μM)	LAPT1 *V*_max_ (pmol/10^7^ cells/s)
STIB 247WT	0.029 ± 0.001	0.008 ± 0.002	14 ± 2	49 ± 19	0.65 ± 0.17
STIB 247MR	NP			56 ± 19	0.41 ± 0.10
STIB 386WT	0.027 ± 0.004	0.007 ± 0.002	22 ± 6	46 ± 9	0.70 ± 0.15
STIB 386MR	NP			51 ± 2	1.2 ± 0.4

Uptake of [^3^H]pentamidine by suspensions of 10^7^ procyclic trypanosomes was measured at 25 nM or 1 μM for the determination of parameters of high-affinity transport (HAPT1 mediated) or low-affinity transport (LAPT1 mediated), respectively. In the 247MR and 386MR strains, no high-affinity pentamidine transport was observed and transport rates were very low, with saturation only at very high concentrations of unlabelled pentamidine, consistent with uptake by LAPT1. NP, not present in these cells.

The characterization of the isogenic pair s427/*TbAT1*-KO clearly showed loss of P2 activity to be associated with only a minor loss of susceptibility to pentamidine and MPAs, in addition to a much higher degree of resistance to diminazene.^14,50^ Thus, high-level MPXR is clearly a function of the loss of *TbAT1* function in addition to other mutation(s).^51^ We investigated MPXR in the *T. b. brucei* 247 and *T. b. gambiense* 386 isogenic pairs, both generated by *in vivo* adaptation to Cymelarsan.^33^ Pentamidine cross-resistance for *T. b. brucei* 247 was first reported by Scott *et al*.^52^ with >50-fold pentamidine resistance *in vivo* for *T. b. brucei* 247MR relative to its parental line and we confirm here that procyclic 247MR were 74- and 755-fold resistant to pentamidine and Cymelarsan, respectively; these numbers were 90- and 83-fold for 386MR (Figure S2, available as Supplementary data at *JAC* Online). Neither strain displayed resistance to phenylarsine oxide (PAO). Assessment of the HAPT1 and LAPT1 transporters in both isogenic pairs confirmed that MPXR was associated with loss of HAPT1 activity: 247MR and 386MR did not express the activity whereas both parental lines did. Figure S3 (available as Supplementary data at *JAC* Online) illustrates this in detail for the 247 isogenic pair and Table 2 summarizes the characterization of HAPT1 and LAPT1 of the 247 and 386 isogenic lines.

We thus analysed the *AQP2-AQP3* locus of the 247 and 386 isogenic pairs and discovered that the *AQP2* gene was completely absent from the 386MR line, whilst the *AQP3* gene was identical to that in the 386 wild-type line. In the 247MR line, however, a chimeric gene of *AQP2*and*AQP3* had been formed in place of both wild-type genes. This chimera is in-frame, producing a 939 bp ORF composed of the first 363 bp of *AQP2* and the last 576 bp of *AQP3*, and is thus different from the chimera found in strain B48 (see below).

Detailed analysis of the AQP locus of the STIB 900 lines revealed that both the pentamidine- and melarsoprol-resistant lines had lost the *AQP2* gene whilst retaining the *AQP3* gene. The organization of the *AQP2-AQP3* locus in all the various strains so far examined is shown in Figure S4 (available as Supplementary data at *JAC* Online). It appears that, in all cases, *AQP2* is either lost completely or recombined into a chimeric gene that encodes most of the residues comprising the AQP3 selectivity filter.

### Expression of AQP2 reverses high levels of pentamidine and melarsoprol resistance

**Figure 1.**
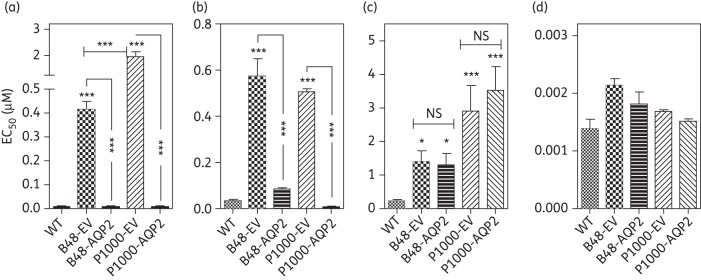
Expression of *TbAQP2* in multidrug-resistant trypanosomes sensitizes to (a) pentamidine and (b) Cymelarsan but not (c) diminazene or (d) phenylarsine oxide. EC_50_ values were obtained using the Alamar blue assay and bars represent the mean and SEM of 3 to >10 independent determinations; data were analysed for significant differences from the wild-type control using one-way ANOVA/Tukey's test (GraphPad Prism 5.0). NS, not significant; *P < 0.05; **P < 0.01; ***P < 0.001; unless otherwise indicated, relative to wild-type controls. EV, empty-vector control.

We used the laboratory-generated cell line B48^16^ and its derivative P1000 to investigate whether expression of wild-type (WT) TbAQP2 can fully reverse the multidrug resistance phenotype of these clones. P1000 was developed by further adaptation of B48 to 1 μM pentamidine *in vitro* and thus its resistance phenotype is believed to be multifactorial; B48 itself was derived from the *tbat1^−/−^* strain and additionally lacks HAPT1 activity.^16^ Figure 1 shows the resistance profile of WT s427 and of B48 and P1000 transfected with the empty vector (EV) pHD1336 and with the same vector containing WT *TbAQP2*.^37^ The lipophilic arsenical PAO was used as a control as it has been shown to diffuse across the *T. b. brucei* plasma membrane,^16^ making its action independent of transporters and showing that the resistance phenotype is not to arsenic *per se*.

The EV controls B48 and P1000 were strongly resistant to the diamidines pentamidine and diminazene as well as to the MPA drug Cymelarsan, but not to PAO. Expression of TbAQP2 in B48 and P1000 completely reversed resistance to pentamidine (50- and 240-fold, respectively) and Cymelarsan (16.7- and 15.0-fold, respectively) in both resistant clones (Figure 1). However, the level of diminazene resistance in these lines was not affected, consistent with the lack of diminazene resistance in an *aqp2* null line.^20^

### A chimeric AQP2/3 gene in the AQP2 locus of B48 and P1000 is distributed over the cell surface and does not affect pentamidine and arsenic sensitivity

In B48, the *AQP2* locus has been replaced by a chimeric in-frame fusion of *AQP2* and *AQP3* (*chAQP2/3*) whereas *AQP3* has remained unchanged.^20^ An identical chAQP2/3 and AQP3 locus was found in P1000, showing that the higher level of pentamidine resistance in P1000 was not due to further changes to the AQP2-AQP3 locus.

**Figure 2.**
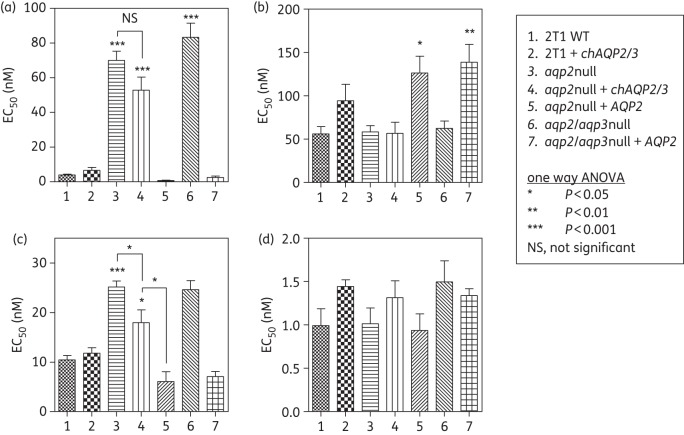
Expression of *TbAQP2* and *chAQP2/3* in *aqp2* null and *aqp2/aqp3* null trypanosomes. EC_50_ values were determined for (a) pentamidine, (b) diminazene, (c) Cymelarsan and (d) phenylarsine oxide. All data are the mean of ≥10 independent determinations. See legend of Figure 1 for details. Significance was tested relative to the wild-type control unless otherwise indicated.

The observation that expression of WT AQP2 reverses the B48 and P1000 MPXR phenotypes (Figure 1) suggests that *chAQP2/3* coincides with the loss of AQP2 function. This was further investigated by expressing either WT *AQP2* or *chAQP2/3* in the *aqp2* null line produced from this strain.^20^ Expression of WT *AQP2* reversed the *aqp2* null phenotype, whereas expression of *chAQP2/3* had much less effect on sensitivity to any of the drugs tested (Figure 2). This confirms that the chimeric form does not function like AQP2, at least with respect to pentamidine susceptibility (*P* > 0.05), whether it still exercises an AQP-like activity or not. We did observe a small but significant (1.4-fold; *P* < 0.05) difference in Cymelarsan susceptibility between the *aqp2* null clone and the same cells expressing *chAQP2/3*, although the effect was very much smaller than the expression of WT *AQP2* (4.1-fold; *P* < 0.001). The (over)expression of WT *AQP2* in an *aqp2* null or *aqp2/aqp3* double-null background appeared to make the cells slightly less susceptible to diminazene, the reverse of its effect on pentamidine and Cymelarsan sensitivity. There were no significant differences between any of the lines with respect to PAO. Expression of *AQP2* in the WT trypanosomes did not elicit any change in drug susceptibility (Figure 2).

**Figure 3.**
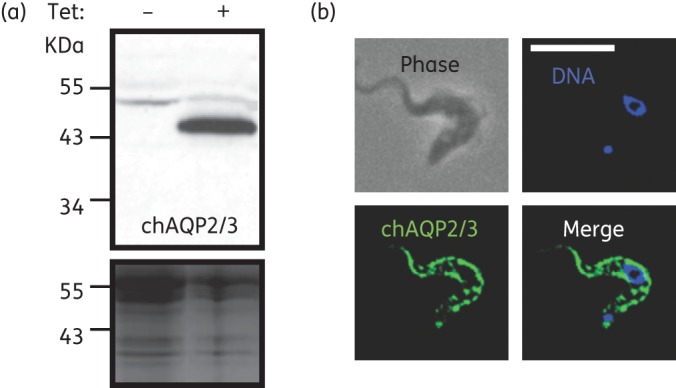
Localization of GFP-chAQP2/3 expressed in bloodstream-form *aqp2/aqp3* null trypanosomes. (a) Western blot using anti-GFP antiserum as described previously.^20^ Blotting was performed after induction with 1 μg/mL tetracycline (+) or without induction as control (−). The Coomassie-stained panel shows relative loading. (b) GFP-chAQP2/3 was localized to the plasma membrane. Blue colour is DAPI staining of nucleus and kinetoplast. The scale bar represents 10 μm.

Apart from the primary sequence differences between *TbAQP2* and *TbAQP3*, their cellular location is also different, with AQP2 restricted to the flagellar pocket whereas AQP3 is present throughout the plasma membrane.^20^ As this could have bearing on the MPXR phenotype, a fusion gene of *chAQP2/3* N-terminally coupled to GFP was constructed and introduced into the *aqp2/aqp3* null strain. Expression of the fusion protein was confirmed by western blotting (Figure 3a). The localization in trypanosomes was observed directly by fluorescence microscopy. It was found that the GFP-tagged chAQP2/3, unlike AQP2, was present on the plasma membrane (Figure 3b).

### Expression of WT TbAQP2 correlates with the HAPT1 pentamidine transport activity

The strong phenotype of pentamidine resistance in *aqp2* null lines (16.3-fold; *n* = 22; *P* < 0.001) prompted us to investigate the uptake of [^3^H]pentamidine in these cells and compare this with that in the parental 2T1 cells. We chose a [^3^H]pentamidine concentration of 30 nM in the presence of 1 mM adenosine; this concentration of adenosine fully blocks the P2 aminopurine transporter^53^ and the 30 nM label concentration produces a biphasic uptake that visualizes uptake through both high- and low-affinity pentamidine transporters (HAPT1 and LAPT1).^16^ Propamidine was used as a specific inhibitor of HAPT1, having no effect on LAPT1 activity.^17^ In four independent experiments in triplicate, [^3^H]pentamidine uptake was assessed, in parallel, in WT and in *aqp2* null cells.

**Figure 4.**
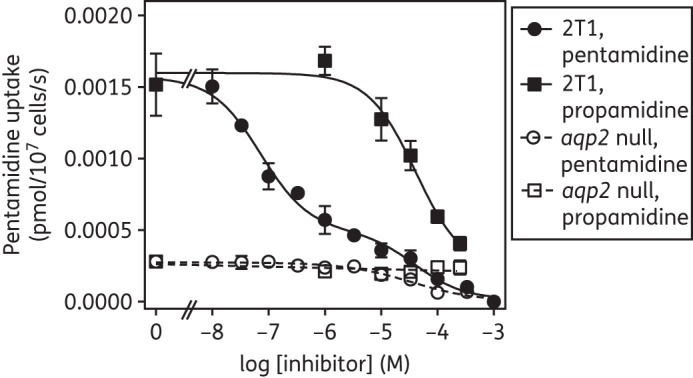
Uptake of 30 nM [^3^H]pentamidine by *aqp2* null and wild-type control cells. Cells of *T. b. brucei* strain 2T1 (closed symbols) or the derived *aqp2* null strain (open symbols) were incubated with [^3^H]pentamidine for 90 s in the presence or absence of various concentrations of unlabelled pentamidine (circles) or propamidine (squares). The incubation was stopped by the addition of 1 mL ice-cold 1 mM pentamidine and immediate centrifugation through oil. The experiment shown is representative of four identical but independent experiments, each performed in triplicate and showing virtually identical outcomes. Data points are mean ± SEM of triplicates.

As expected in control trypanosomes, inhibition of the uptake of [^3^H]pentamidine with unlabelled pentamidine in the range 10 nM–1 mM produced a biphasic curve, of which only the first (high-affinity) phase was sensitive to inhibition by propamidine (IC_50_ = 29.9 ± 4.3 μM; *n* = 3). Plotting the pentamidine inhibition data to an equation for a biphasic sigmoidal curve (two-site inhibition; GraphPad Prism 5.0) yielded mean IC_50_ values that were entirely compatible with the HAPT1/LAPT1 system: 0.060 ± 0.017 and 29.9 ± 4.3 μM (*n* = 3), respectively (Figure 4). In the *aqp2* null cells, [^3^H]pentamidine uptake was much lower, completely lacked the high-affinity inhibition phase with unlabelled pentamidine and was insensitive to propamidine (Figure 4). The mean IC_50_ value of 33.4 ± 9.5 μM was statistically identical to the lower-affinity phase of the WT 2T1 cells and with the published pentamidine *K*_m_ for LAPT1 (56.2 ± 8.3 μM).^17^ We conclude that *aqp2* null cells do not express HAPT1, but do express LAPT1.

**Figure 5.**
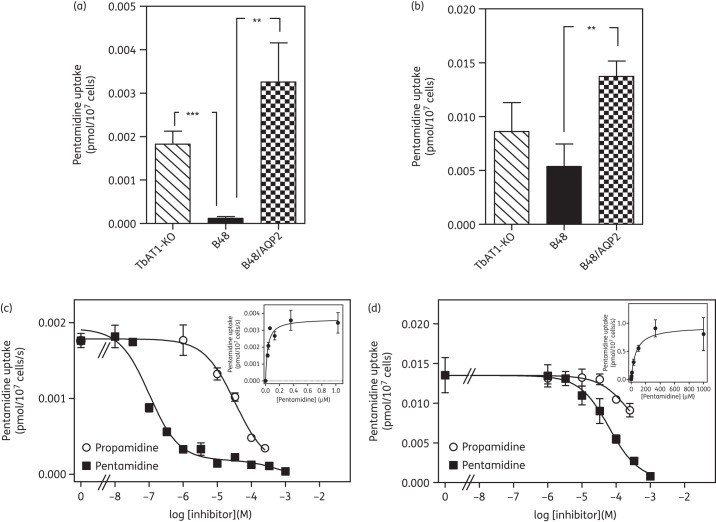
[^3^H]pentamidine uptake by various s427-derived trypanosome lines. (a) [^3^H]pentamidine concentration was 50 nM, reflecting predominantly HAPT1-mediated uptake. (b) [^3^H]pentamidine concentration was 1 μM, reflecting predominantly LAPT1 uptake. Rates were determined from the slopes of time courses over 10 min with timepoints at 0, 1, 3, 5, 7.5 and 10 min. Lines were linear, with none of the lines showing significant deviation from linearity in a runs test (GraphPad Prism 5.0). Slopes of control time courses in the presence of 1 mM unlabelled pentamidine were all non-significantly different from zero (*F*-test, GraphPad Prism 5.0), whereas uptake with unlabelled inhibitor was highly significantly different from zero (typically *P* < 0.0001). Results are the mean of three to four independent experiments, each performed in triplicate. (c, d) Uptake of [^3^H]pentamidine in bloodstream forms of B48/AQP2 was determined as described in the legend to Figure 4. The experiments shown are representative of three independently performed replicates. (c) Uptake of 30 nM [^3^H]pentamidine. (d) Uptake of 1 μM [^3^H]pentamidine. Solid squares, coincubation with various concentrations of unlabelled pentamidine; open circles, coincubation with propamidine. **P < 0.01; ***P < 0.001.

We previously reported that the pentamidine-resistant clone B48 similarly lacks HAPT1 pentamidine transport activity as a result of *in vitro* resistance selection with pentamidine^16^ and that this clonal line lacks a WT *AQP2* gene.^20^ We therefore tested whether introduction of wild-type *TbAQP2*, in addition to restoring pentamidine susceptibility (Figure 1), would reinstate HAPT1 activity. Figure 5 shows the rates of uptake at [^3^H]pentamidine concentrations that favour uptake by HAPT1 (50 nM; Figure 5a) or LAPT1 (1 μM; Figure 5b); an s427-based cell line from which the *TbAT1*/P2 transporter was removed by homologous recombination (*TbAT1*-KO)^14^ was used as the control, as this was the strain B48 was derived from.^16^ Consistent with earlier findings, uptake of 50 nM [^3^H]pentamidine was reduced 15.5-fold in B48 compared with in the *TbAT1*-KO control (*P* < 0.001), whereas uptake of 1 μM pentamidine was not significantly different. Expression of *TbAQP2* in B48 increased uptake of 50 nM pentamidine 27.7-fold (*P* < 0.02; Figure 5a) to a level that appeared somewhat higher than that in *TbAT1*-KO, although that difference did not reach statistical significance. Uptake of 1 μM pentamidine was also increased upon introduction of *TbAQP2*, but by only 2.5-fold (*P* < 0.02; Figure 5b) and again was not significantly different from the level in *TbAT1*-KO. These data confirm that expression of *TbAQP2* in B48 restores the pentamidine transport conditions of *TbAT1*-KO, completely reversing transport-related resistance.

We next investigated whether the increased pentamidine uptake rates were indeed due to the expression of a HAPT1 activity in B48 cells re-expressing AQP2. *K*_m_ and *V*_max_ values of HAPT1 and LAPT1 were determined in B48/AQP2 (Figure 5c and d) and compared with previously obtained values for WT s427, B48 and *TbAT1*-KO (Table 3). No clear or significant differences were observed with the kinetic parameters of pentamidine transport in bloodstream forms of other s427-derived strains (WT s427, TbAT1-KO and B48), including P1000, for which the pentamidine *K*_m_ and *V*_max_ values (Figure S5; available as Supplementary data at *JAC* Online) were also added to Table 3. We conclude that expression of TbAQP2 reinstated a high-affinity pentamidine transport activity that is indistinguishable from the well-characterized HAPT1, whilst making no detectable change to the LAPT1 activity.

**Table 3.** Kinetic parameters of high-affinity and low-affinity pentamidine transport in various strains of *T. b. brucei* bloodstream forms and in *Leishmania mexicana* expressing TbAQP2

Strain	HAPT1 pentamidine *K*_m_ (μM)	HAPT1 propamidine *K*_i_ (μM)	HAPT1 diminazene *K*_i_ (μM)	LAPT1 pentamidine *K*_m_ (μM)
WT s427^a^	0.036 ± 0.006	4.6 ± 0.7	63 ± 3	56 ± 8
TbAT1-KO^b^	0.029 ± 0.008	13.0 ± 3.0	ND	50 ± 17
B48^c^	NP			56 ± 7
P1000	NP			99 ± 24
B48/AQP2	0.046 ± 0.014	15.2 ± 1.6	ND	66 ± 1
*L. mexicana*/TbAQP2	0.055 ± 0.004	8.1 ± 0.8	100 ± 21	not present in these cells

ND, not determined; NP, not present in these cells.

In the B48 and P1000 strains, no high-affinity transport of pentamidine was detectable, nor was pentamidine uptake inhibited by propamidine.

^a^De Koning.^17^

^b^Matovu *et al*.^14^

^c^Bridges *et al*.^16^

### TbAQP2 displays HAPT1 activity when expressed in L. mexicana

**Figure 6.**
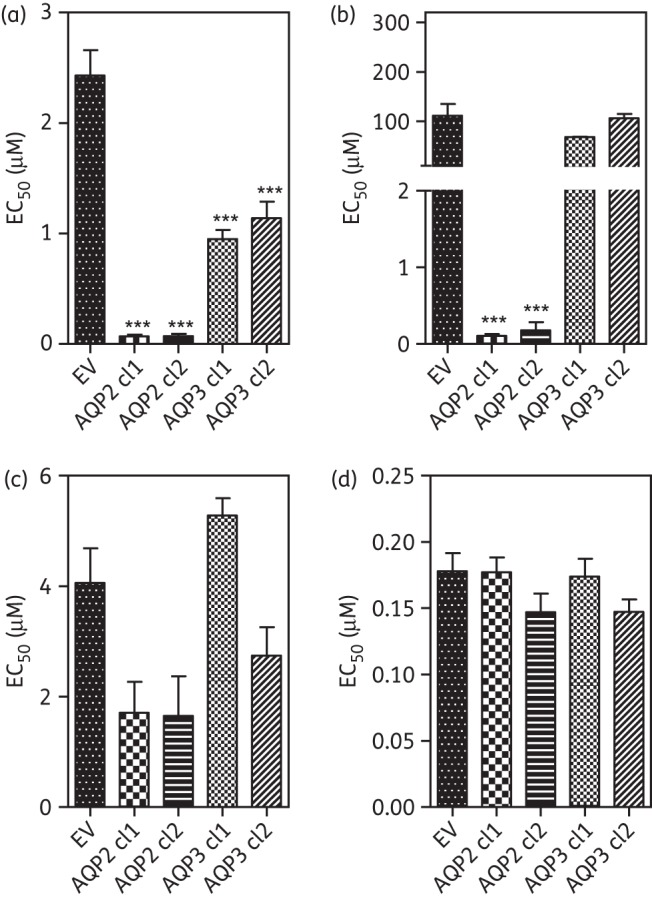
Effect of expression of *T. brucei* aquaporins on drug susceptibility of *Leishmania mexicana*. TbAQP2 and TbAQP3 were expressed in promastigotes using the pNUS vector. Two independent clones of each resulting cell line and the promastigotes transfected with the ‘empty’ pNUS vector were tested for sensitivity to (a) pentamidine, (b) Cymelarsan, (c) diminazene and (d) amphotericin B using the Alamar blue fluorimetric assay. Bars are means of three to eight independent determinations; error bars are SEM. ***P < 0.001 by one-way ANOVA with Tukey's correction (GraphPad Prism 5.0). EV, empty-vector control.

*Leishmania* spp. are relatively insusceptible to pentamidine and tolerant to MPAs, as they lack high-affinity uptake systems for these drugs. In order to investigate whether *T. b. brucei* AQPs directly mediate uptake of these drugs, we expressed *TbAQP2* and *TbAQP3* in promastigotes of *L. mexicana*. Comparisons of control cells (transfected with EV) and cells expressing *TbAQP2* showed 40-fold sensitization in two independent clones (Figure 6a) and a >1000-fold sensitization to MPAs (Figure 6b). There was no statistically significant effect on sensitivity to diminazene, relative to the EV control (Figure 6c), or to amphotericin B (Figure 6d); the latter was used as a control antileishmanial drug thought to bind to plasma membrane ergosterol, thereby forming pores.^54^ Expression of *TbAQP3* in two independent clones led to minor (1.5–2.5-fold) sensitization to pentamidine.

**Figure 7.**
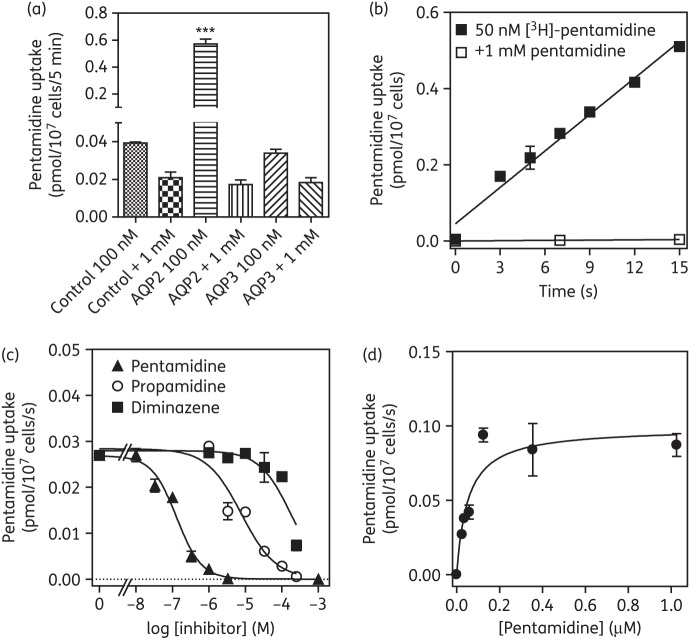
Expression of *T. b. brucei* aquaporins in promastigotes of *Leishmania mexicana*. (a) Specific uptake of 100 nM [^3^H]pentamidine over 5 min in *L. mexicana* promastigotes transfected with empty pNUS vector (control), or promastigotes transfected with *TbAQP2* or with *TbAQP3*. In each case, mediated uptake of 100 nM radiolabel was compared with total association of [^3^H]pentamidine with the cell pellet in the presence of a saturating concentration (1 mM) of unlabelled pentamidine. The data shown are the mean of triplicates ± SEM. ***P < 0.001 by one-way ANOVA, compared with all other datasets. (b) Time course of 50 nM [^3^H]pentamidine uptake, over 15 s, using *L. mexicana* promastigotes transformed with *TbAQP2* in the presence and absence of 1 mM unlabelled pentamidine. Uptake at 50 nM pentamidine was linear (*r*^2^ = 0.98) and rapid (0.032 ± 0.002 pmol/10^7^ cells/s, compared with 0.00026 ± 1.8 × 10^−6^ pmol/10^7^ cells/s in the presence of 1 mM pentamidine). (c) Characterization of 20 nM [^3^H]pentamidine uptake in *L. mexicana* promastigotes expressing TbAQP2, in the presence of varying concentrations of unlabelled inhibitor. (d) Michaelis–Menten plot of 20 nM [^3^H]pentamidine uptake; conversion of pentamidine inhibition plot in (c).

Consistent with this minor effect of AQP3 expression, uptake of 100 nM [^3^H]pentamidine in *TbAQP3*-transfected promastigotes was not significantly different from that in the control when measured over 5 min (Figure 7a and data not shown). In contrast, pentamidine uptake in *TbAQP2*-expressing cells was increased almost 15-fold (Figure 7a). Indeed, uptake of very low concentrations of [^3^H]pentamidine (50 nM) was measurable in *TbAQP2*-expressing promastigotes within 3 s, was linear with a rate of 0.032 ± 0.002 pmol/10^7^ cells/s and completely saturable with excess unlabelled pentamidine (Figure 7b). Competition with unlabelled pentamidine, propamidine and diminazene was dose dependent (Figure 7c) and followed Michaelis–Menten kinetics (Figure 7d). Mean *K*_m_ and *K*_i_ values for these inhibitors are listed in Table 3 and show a kinetic profile entirely consistent with the *T. b. brucei* HAPT1 activity, with an apparent *K*_m_ value of 55 ± 4 nM (*n* = 5).

### TbAQP2 does not affect the activity of the P2 transporter

It has long been established that the P2 transport activity, encoded by *TbAT1*, is an important determinant of diamidine and arsenical sensitivity in *T. brucei*.^8,12^ It is thus important to establish whether the TbAQP2/HAPT1 activity has any effect on P2 activity. It has been previously reported that the expression of P2 can be assessed through susceptibility to some adenosine analogues.^55^ Indeed, we found that susceptibility to tubercidin (7-deazaadenosine) decreased 10-fold in the *tbat1^−/−^* strain (EC_50_ = 2.65 ± 0.23 μM) relative to s427 WT (0.23 ± 0.03 μM; *P* < 0.001; Student's *t*-test). Similarly, susceptibility to tubercidin, cordycepin (3′-deoxyadenosine) and 5′-deoxyadenosine increased >20-fold when *TbAT1* was expressed in the *tbat1^−/−^* strain B48 (*P* < 0.001) (Figure S6; available as Supplementary data at *JAC* Online), confirming susceptibility to cytotoxic adenosine analogues as a sensitive indicator for *TbAT1* expression levels. However, the susceptibility of 2T1 WT and *aqp2* null cells to these same adenosine analogues was identical, as was the susceptibility of B48 + *TbAQP2* versus control B48 cells (Figure S6, available as Supplementary data at *JAC* Online). Susceptibility to pentamidine, used as a positive control, was highly dependent on the expression of *TbAQP2* and/or *TbAT1*. We conclude that TbAQP2 does not regulate P2 activity and is not itself a conduit for cytotoxic nucleosides.

### Investigation of the TbAQP2 and TbAQP3 selectivity filters as the determinant for MPXR

**Figure 8.**
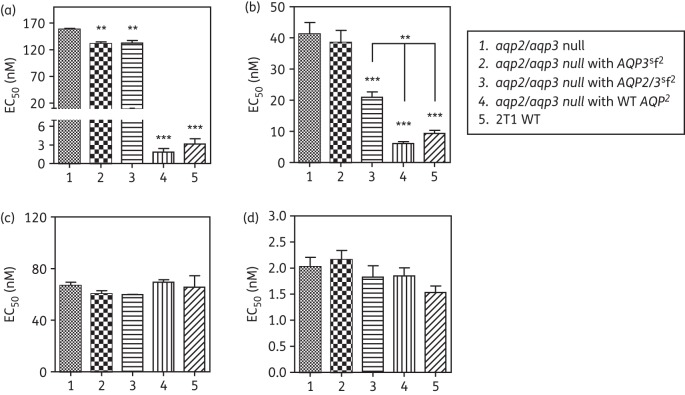
Expression of synthetic aquaporin constructs in bloodstream forms of the *aqp2/aqp3* double-null line. EC_50_ values were determined for (a) pentamidine, (b) Cymelarsan, (c) diminazene and (d) phenylarsine oxide. Expression was induced by the addition of 1 μg/mL tetracycline 24 h before setting up the plates for the assay, followed by 48 h of incubation of the cells in the presence of a doubling dilution of the test compound followed by a further 24 h of incubation with the dye before fluorescence was determined. All data are the mean of at least three independent determinations; error bars are SEM. Statistical significance with *aqp2/aqp3* null was determined using a one-way ANOVA with Tukey's correction (GraphPad Prism 5.0); **P < 0.02; ***P < 0.01. In the Cymelarsan panel (b), it is also indicated that Groups 4 and 5 are significantly different from Group 3.

The *TbAQP2* region that was replaced with the corresponding *TbAQP3* sequence in the recombination event that generated *chAQP2/3* contains most of the selectivity filter that is believed to determine the distinct permeation profiles of AQPs.^20^ As the chimeric AQPs found in B48 and 247MR did not appear to have the TbAQP2 functionality with respect to pentamidine and melarsoprol susceptibility, we investigated whether MPXR is determined principally by the few amino acids of the selectivity filter. We used synthetic genes of *chAQP2/*3 and of *TbAQP3* with a *TbAQP2* selectivity filter (*chAQP2/3*^sf2^ and *AQP3*^sf2^, respectively; alignment in Figure S7, available as Supplementary data at *JAC* Online). These were expressed in the *aqp2/aqp3* null cell line. Analysis of the drug susceptibility phenotype for the resultant lines showed that the transplantation of the amino acid residues of the AQP2 selectivity filter did not result in an AQP2 phenotype with respect to drug susceptibility; there was only a minor increase in susceptibility and only to pentamidine (EC_50_ values of 158 ± 2 and 131 ± 3 nM, *P* < 0.02, for *aqp2/aqp3* null and *AQP3*^sf2^, respectively; Figure 8). In contrast, the cell line expressing the *chAQP2/3*^sf2^ construct was significantly more susceptible to Cymelarsan than the *aqp2/aqp3* null background, reaching susceptibility to this drug halfway between the *aqp2/aqp3* null and the same line expressing WT AQP2 (Figure 8). These findings indicate that the selectivity filter residues do play a role in arsenical drug sensitivity, but that the changes described here were insufficient to produce the same effect as WT AQP2 on pentamidine susceptibility.

## Discussion

Although we recently reported that the absence of a wild-type *AQP2* gene correlates with MPXR in *T. b. brucei*,^20^ many important questions remained. It remained unclear (i) whether this phenomenon is relevant for human-infective trypanosome subspecies, (ii) whether loss of *AQP2* occurs as a result of *in vivo* drug exposure, (iii) whether loss of *AQP2* alone is sufficient for high levels of pentamidine and melarsoprol resistance, (iv) by which mechanism *AQP2* facilitated pentamidine and melarsoprol sensitivity and (v) whether *AQP2* has any impact on *TbAT1*/P2 activity. We now provide answers to these questions.

Only TbAQP2 was implicated in MPXR, with the expression of TbAQP3 in an *aqp2/aqp3* null line having no effect on drug susceptibility.^20^ This is most likely the result of differences in permeation, as all three TbAQPs are located on the cell surface, although AQP2 was found to be restricted to the flagellar pocket rather than dispersed over the plasma membrane.^20,28^ Interestingly, a chimeric AQP, made up from *TbAQP2* and *TbAQP3* (*chAQP2/3*) was present instead of wild-type *TbAQP2* in the highly MPXR strain *T. b. brucei* B48, which retained a wild-type copy of *TbAQP3* found in the parental, drug-susceptible strain.^20^ Here, we report that in other isogenic MPXR pairs, the adapted strain similarly showed a replacement of *TbAQP2* with a different chimeric *AQP2/3* fusion or an outright deletion of *TbAQP2*. This was the case whether the strain was originally adapted to pentamidine or to an MPA drug, whether the subspecies was *T. b. brucei*, *T. b. gambiense* or *T. b. rhodesiense* and for strains that were selected by either *in vivo* or *in vitro* drug pressure. It thus follows that for *T. brucei* subspecies, loss of TbAQP2 function represents an important component of acquiring high levels of resistance to MPAs and that this necessarily leads to pentamidine resistance as well, and vice versa. We also conclude that loss of AQP2 can be the result of *in vivo* drug pressure and that this does not significantly affect virulence and does not impede transmission by tsetse flies, as STIB 386MR and STIB 247MR produce similar parasitaemias in mice compared with the wild-type lines from which they were derived and were fly transmissible.^33^

One important question is whether loss of *TbAQP2* alone is sufficient for MPXR *in vivo*. Certainly, the deletion of just *TbAQP2* rendered a highly drug-susceptible *T. b. brucei* strain resistant to pentamidine (but not diminazene) and Cymelarsan (but not PAO) *in vitro*;^20^ this shows that the resistance is not to all aromatic diamidines or to arsenic *per se*. Similarly, the reintroduction of wild-type *TbAQP2* into B48 or P1000 restored sensitivity to Cymelarsan and pentamidine (but not diminazene) to wild-type levels. Both strains retained their diminazene resistance, which is linked to the loss of the *TbAT1*/P2 transporter,^14,50^ which is absent in B48 and P1000 cells.^16^ Indeed, *TbAT1* is absent or mutated in all the drug-adapted MPXR strains used here,^56,57^ opening up the possibility that both transport activities must be absent for high levels of MPXR. It has already been established that homozygous deletion of *TbAT1* alone results in a relatively minor (2.5–3-fold) loss of sensitivity to pentamidine and MPAs;^14^ a similar level of MPA resistance was observed in the *aqp2* null and *aqp2*/*aqp3* null lines, which do retain a wild-type copy of *TbAT1*. Although the data presented here and by Baker *et al*.^20^ show that the deletion of *TbAQP2* alone is sufficient to give a strong pentamidine resistance phenotype *in vitro* (17.5-fold; Figure 2), it is worth exploring this in detail. Firstly, *TbAT1*/P2 activity is very low in cultured *T. b. brucei* bloodstream forms, compared with expression *in vivo*,^15^ dramatically reducing the influence of this transporter on drug sensitivity. Secondly, the pHD1336 vector used to reintroduce *TbAQP2* in B48 and P1000 gives a very robust expression level,^37^ well above that in the wild-type, which helps explain the complete reversal of MPA/pentamidine resistance even in the absence of *TbAT1*. Finally, the reintroduction of *TbAT1* in B48 in the same vector also led to an almost complete reversal of resistance to pentamidine, Cymelarsan and diminazene, even in the continued absence of a wild-type *TbAQP2*.^58^

The conclusion from the above must be that loss of either *TbAQP2* or *TbAT1* activity can lead to some loss of MPA and pentamidine susceptibility, but that the high MPXR phenotype is the result of both being lost concomitantly. B48, which lacks both genes, is more resistant to pentamidine and MPAs than either *aqp2* null or the *TbAT1* knockout strain from which it was derived. Thus, the combined data strongly suggest that TbAQP2 is the previously described HAPT1 and transports MPAs and pentamidine. A number of observations reported in the present article strongly support this notion: (i) the *T. b. brucei* strains B48, P1000 and 247MR and *T. b. gambiense* 386MR have all lost HAPT1 activity and also lack a wild-type copy of *TbAQP2*; (ii) targeted deletion of *TbAQP2* specifically removes the high-affinity pentamidine transport component, leaving the low-affinity transport activity unchanged; (iii) expression of *TbAQP2* in B48 restores HAPT1 activity; (iv) expression of *TbAQP2* in *L. mexicana* causes a massive sensitization to pentamidine and Cymelarsan; and (v) the introduction of a high-affinity pentamidine transporter that is indistinguishable from HAPT1 by *K*_m_ value and inhibitor profile. Thus, the evidence overwhelmingly supports a model that TbAQP2 mediates the saturable uptake of pentamidine and MPAs, following standard Michaelis–Menten kinetics, in addition to its more conventional function as a water/glycerol channel.

As discussed by Baker *et al*.,^20^ TbAQP2 has a number of unusual residues in the motifs that are believed to be involved in selectivity: NPS/IVLL is replaced with the classical regions of NPA/IGYR in the chimera. To begin to unravel the structural determinants of AQP2 action, we synthesized two new genes, with the NPS/IVLL motif transplanted to either *chAQP2/3* or *TbAQP3*, and expressed these constructs in an *aqp2/aqp3* null strain. The resulting cell lines displayed slightly higher pentamidine sensitivity than the control and the cells expressing *chAQP2/3*^sf2^ showed a substantial sensitization to Cymelarsan. It follows that the usual selectivity filter of TbAQP2 does indeed contribute to its drug transport capacity, but is not the sole determinant.

In summary, we have shown that an aquaglyceroporin, TbAQP2, mediates the saturable uptake of some first-line trypanocides, Cymelarsan and pentamidine, compounds of substantially higher molecular weight than has so far been reported for any AQP. The mechanism by which this MIP transports these drugs with Michaelis–Menten kinetics remains to be investigated.

## Funding

This work was funded by a grant to H. P. dK., M. P. B. and R. J. S. B. from the Medical Research Council (84733) and a grant to D. H. from The Wellcome Trust (093010/Z/10/Z) and by the Medical Research Council (MR/K000500/1). D. H. is also supported by a Wellcome Trust Senior Investigator Award (100320/Z/12/Z) and by the Medical Research Council (MR/K000500/1). A. A. E. was supported by a British Commonwealth scholarship. N. B. was supported by a Bloomsbury Colleges studentship. F. E. G. was supported by the Swiss National Science Foundation (31003A_135746), P. L. by the Emilia Guggenheim-Schnurr Foundation, the Freiwillige Akademische Gesellschaft Basel, and the Mathieu Foundation of the University of Basel.

## Transparency declarations

None to declare.

## Supplementary data

Table S1 and Figures S1–S7 are available as Supplementary data at *JAC* Online (http://jac.oxfordjournals.org/).

Supplementary Data
