# Supplementary material for: Trypanosoma brucei aquaglyceroporin 2 is a high-affinity transporter for pentamidine and melaminophenyl arsenic drugs and the main genetic determinant of resistance to these drugs
Source: J Antimicrob Chemother. 2013 Nov 13;69(3):651–63. doi: 10.1093/jac/dkt442 (PMC3922157; doi:10.1093/jac/dkt442)
Supplement: Supplementary Data [file supp_69_3_651__index.html]

Trypanosoma brucei aquaglyceroporin 2 is a high-affinity transporter for pentamidine and melaminophenyl arsenic drugs and the main genetic determinant of resistance to these drugs — Trypanosoma brucei aquaglyceroporin 2 is a high-affinity transporter for pentamidine and melaminophenyl arsenic drugs and the main genetic determinant of resistance to these drugs — Supplementary Data 

# *Trypanosoma brucei* aquaglyceroporin 2 is a high-affinity transporter for pentamidine and melaminophenyl arsenic drugs and the main genetic determinant of resistance to these drugs

## Supplementary Data

Supplementary Data

**Files in this Data Supplement:**

- Supplementary Data - Docx file
