# Supplementary material for: Trypanosoma brucei aquaglyceroporin 2 is a high-affinity transporter for pentamidine and melaminophenyl arsenic drugs and the main genetic determinant of resistance to these drugs
Source: J Antimicrob Chemother. 2013 Nov 13;69(3):651–63. doi: 10.1093/jac/dkt442 (PMC3922157; doi:10.1093/jac/dkt442)
Supplement: Supplementary Data [file supp_dkt442_dkt442supp.docx]

Supplementary data

Table S1

| **Primer** | **Sequence 5'-3'** | **Restriction Site** | **Gene** |
| --- | --- | --- | --- |
| HDK208 | CGGGCCCATGCAGAGCCAACC | *Apa*I | Forward AQP2 / AQP2-3 |
| HDK209 | CCCGATCCTTAGTGTGGAAGAAA | *Bam*HI | Reverse AQP2 / AQP2-3 |
| HDK147 | CGTCACGCAAGGTGCTTTGC |  | Forward AQP2 in P1000 / 247-WT  and Forward AQP3 in 247-WT / 247-Mr |
| HDK160 | CCTATTCTATCGCCCGCTGC |  | Forward AQP2 P1000 |
| HDK146 | CCACTAACCGTCAATACCC |  | Reverse AQP2 in P1000 / 386-WT / 247-WT |
| HDK324 | GATATTTACAACGGGAGGTC |  | Forward AQP2 in 386-WT |
| HDK144 | CCCCTTGCGTTTTACCTTTGC |  | Reverse AQP3 in 386-WT / 386-Mr / 247-WT / 247-Mr and Reverse AQP2-3 in 247-Mr |
| HDK145 | GGCTGAAACTCCACTTGTTG |  | Forward AQP3 in 386-WT / 386-Mr and Forward AQP2-3 in 247-Mr |
| HDK327 | CATATGCAGAGCCAACCAGAC | *Nde*I | Forward AQP2 / AQP3 |
| HDK328 | CGAGATCTTAGTGTGGAAGAAA | *Bgl*II | Reverse AQP2 |
| HDK329 | CGAGATCTTAGTGTGGCACAAA | *Bgl*II | Reverse AQP3 |
| HDK157 | CCCGAGAAGGATCGCACCG |  | Reverse AQP2/pNUS |
| HDK159 | GCCGGATTTATTGCCTAACC |  | Reverse AQP3/pNUS |
